# We know but we hope: A qualitative study of the opinions and experiences on the inclusion of management, health economics and research in the medical curriculum

**DOI:** 10.1371/journal.pone.0276512

**Published:** 2022-10-21

**Authors:** Astrid Turner, Mandy Ryan, Jacqueline Wolvaardt

**Affiliations:** 1 School of Health Systems and Public Health, Faculty of Health Sciences, University of Pretoria, Pretoria, South Africa; 2 Health Economics Research Unit, University of Aberdeen, King’s College, Aberdeen, Scotland; University of Cape Town, SOUTH AFRICA

## Abstract

The achievement of global and national health goals requires a health workforce that is sufficient and trained. Despite considerable steps in medical education, the teaching of management, health economics and research skills for medical doctors are often neglected in medical curricula. This study explored the opinions and experiences of medical doctors and academic educationalists on the inclusion of management, health economics and research in the medical curriculum. A qualitative study was undertaken at four medical schools in Southern Africa (February to April 2021). The study population was medical doctors and academic educationalists. Semi-structured interviews with purposively sampled participants were conducted. All interviews were recorded and professionally transcribed. Constructivist grounded theory guided the analysis with the use of ATLAS.ti version 9.1.7.0 software. In total, 21 academic educationalists and 28 medical doctors were interviewed. In the first theme We know, participants acknowledged the constraints of medical schools but were adamant that management needed to be taught intentionally and explicitly. The teaching and assessment of management and health economics was generally reported to be ad hoc and unstructured. There was a desire that graduates are able to use, but not necessarily do research. In comparison to management and research, support for the inclusion of health economics in the curriculum was insignificant. Under We hope, educationalists hoped that the formal clinical teaching will somehow instil values and best practices of management and that medical doctors would become health advocates. Most participants wished that research training could be optimised, especially in relation to the duration of allocated time; the timing in the curriculum and the learning outcomes. Despite acknowledgement that management and research are topics that need to be taught, educationalists appeared to rely on chance to teach and assess management in particular. These qualitative study findings will be used to develop a discrete choice experiment to inform optimal curricula design.

## Introduction

Although health outcomes such as life expectancy, maternal mortality and others have improved in South Africa, in comparison to similar countries with far less resources, the rate and magnitude of these improvements in South Africa have not been commensurate with the economic investment in the health sector. The most recent national health strategy to address issues concerning the health workforce states that one of the strategic implications highlighted by the country’s complex, multiple burden of disease is that the health professional training and development platform must provide for this wide spectrum of conditions [1]. This fundamental step is also needed for the achievement of the sustainable development goals (SDG), in particular SDG 3, that aims to ensure healthy lives and promote well‐being for all at all ages, and requires not only sufficient numbers of healthcare workers but also ones that are trained to improve health outcomes [2]. A published comment aptly entitled “Health-care workers as agents of sustainable development” highlighted that “….decades of evidence from countries of all income levels speak to the fact that investing in recruitment, retention, and high-quality development and training of health-care workers brings ample short-term and long-term returns and has a high opportunity value for other sectors’ performance” [3].

The shortage of health professionals–in particular medical doctors–is not being addressed adequately in South Africa [1, 4–6]. Prior to the COVID-19 pandemic, there were growing concerns in similar countries about the capacity, quality and productivity of the health workforce where the production of these medical doctors is costly, time-consuming and where curricula content has been informed historically [7–10]. The pandemic has exacerbated and accelerated the shortfall of frontline workers through multiple mechanisms, including the associated mortality rates, mental health impact and pressures to participate in task teams or assume managerial roles [11].

In 2010, the Lancet Commission on transformative learning and education called for “…specific competencies, not time or academic turf protection, must be the defining feature of the education and evaluation of future health professionals”. [12] Since the Commission, there has been some consensus on curriculum content in medical education, albeit with differences in emphasis [13, 14]. However, there is acknowledgement that clinical skills do not translate directly into the capacities required of managers, leaders and change agents needed to navigate the health system, alluding to gaps in the medical training [15–17].

In Southern Africa, the transformation of education needed to produce sufficient medical doctors for complex healthcare needs and systems and meet the regulatory requirements of educational statutory bodies, remain constrained by increasing class sizes, limited training health facility platforms and diminished academic capacity to teach and provide service delivery [7, 12, 18–20].

Specific competencies such as critical thinking, competencies related to leadership and management and an acute awareness of the resource-constrained economic environment may be either neglected as formal teaching in curricula, or if taught, they are inappropriately placed or named or limited in terms of teaching time and content [21–25]. The teaching of management, health economics and research has been restricted in undergraduate medical curricula that have many discipline-specific subjects that compete for time [26–33].

The last decade of medical education has focussed on the development of management and leadership capability to not only prepare graduates for more senior managerial roles but also ensure that clinical practitioners are trained to contribute to organization of care to ensure successful patient care and health services outcomes [15, 34–37]. However, these skills are rarely given sufficient attention in undergraduate curricula and individuals who wish to pursue leadership and management training may need to do so at their own expense and/or develop these skills on the job. All too often the clinical and/or research qualifications and experiences of a doctor are equated to their leadership and management skills [38]. Since there has often been no, or limited, structured learning opportunities and application of these skills in undergraduate training, this leads to what is termed as a “double loss”; the removal of a highly skilled clinician from a clinical role and the instalment of a leader who may not be adequately prepared or experienced in leading a complex health care organization [38].

There have been substantial efforts to include training on health economics in the medical curricula of some institutions as well as developing online resources to support health economics teaching [39, 40]. Short commentaries and studies have highlighted different aspects of health economics in an undergraduate medical curriculum; either focussing on the unrealistic expectations of junior health professionals to provide “high-value, cost-conscious care” if they are not equipped with the basic economic tools or the disproportionate attention that is given to it in comparison to clinical content [21, 24, 27, 28, 41].

Finally, in recognition of the importance of research training such as developing critical thinking and problem-solving attributes, collaborative skills for teamwork and the ability to appraise, and interpret and apply evidence, most medical curricula do include formal training on basic research skills. However, the inclusion is often restricted by limited teaching time, the allocation of credits and the availability of appropriate mentors or supervisors.

Therefore, this study explored the opinions and experiences of medical doctors and academic educationalists on the inclusion of management, health economics and research in the medical curriculum in four settings in South Africa and Botswana.

## Methods

A sequential mixed-methods study design was used whereby the findings of the qualitative research phase (reported in this article) was used to build a quantitative tool to inform optimal curricula design (not reported in this article). The qualitative phase was undertaken from February to April 2021 at four medical school study sites (SS). Three of these sites are in South Africa and one is in Botswana. The sites were selected based on differences in their medical curricula programmes (e.g. when the medical school was established and the model of the programme) as well as location.

After obtaining ethical approval from all SSs, (Health Research Ethics Committee, Faculty of Health Sciences, University of Pretoria: 277/2020; Sefako Makgatho Health Sciences University: 277/2020; Health Research Ethics Committee, Stellenbosch University: S20/06/152 and the Office of Research and Development, University of Botswana Institutional Review Board: UBR/RES/IRB/BIO/GRAD/218), virtual or face-to-face (observing COVID-19 precautions) interviews with medical doctors and academic educationalists who had insight into the curriculum design process were conducted. Written informed consent was obtained for the participation and audio-recording of all interviews.

Academic educationalists were purposively sampled based on their role and responsibilities in the undergraduate medical curriculum. At some sites, this group included the Dean, School of Medicine Chairperson, the Deputy Deans of Research as well as Teaching and Learning (or their equivalents) and other academics who were part of curriculum development. Each of the identified staff were invited to be interviewed. Five to eight participants per SS were interviewed using a semi-structured questionnaire and descriptor cards that outlined the definitions of management, health economics and research to ensure mutual understanding of the terms.

A snowballing sampling strategy was used for the selection of medical participants who were registered medical doctors and alumni of the sites (5 to 11 per SS). Medical graduates that the researcher and supervisor knew through other platforms were contacted and invited to participate. They were also requested to provide contact details for colleagues who they knew graduated from one of the study sites, were registered medical doctors and provided their consent to pass on their contact details. Although descriptor cards were also used, the interview guide for the doctors focused on their personal experiences while the interview guide for the educationalists focused on the current educational strategy at their institution. Upon informed consent, all interviews were recorded or audiotaped. The interviews were professionally transcribed.

The quality of the study can be influenced by the expertise of the researchers, methodological congruence and procedural precision [42]. In terms of the first factor, the first author is an experienced medical doctor with postgraduate qualifications in public health and health economics. She is also an educationalist, having completed a health professions education fellowship programme and actively participates in academic teaching and learning committees and initiatives, including that of curriculum design. In addition, the second author is a health economist whose discipline requires preparatory work using qualitative research methods, which keeps her acquainted with the discipline. The third author is an established educationalist with a doctorate in health professions curriculum development and instructional design, and a public health professional with expertise in qualitative research.

Methodological congruence was attained between the pragmatic worldview that was adopted for the mixed-methods study design [43]; the aim of the research and the constructivist grounded theory approach that was used during the six phases of the thematic analysis from Braun and Clarke (2006), which is summarised in Table 1 [42, 44].

**Table 1:** Phases of thematic analysis adapted for the study.

Phase	Description of the process
1. Familiarization with the data	Reading and re-reading the transcripts per study site and then with the transcripts categorised as doctors and educationalists. Notes of initial thoughts were made.
2. Generation of initial codes	Coding and re-coding of the data as collected and also linking appropriate quotations to them. The second co-investigator worked closely with the principal investigator on iterative rounds of coding and development of categories, sub-themes and the final themes, manually and using ATLAS.ti version 9.1.7.0 software [44]. At regular intervals, these were shared with the first co-investigator for review and discussion by all.
3. Search for themes	Continuing with intermediate coding by collating codes into potential categories towards theory development and thereafter exploring potential sub-themes and themes.
4. Review of themes	Generating a thematic ‘map’ of the analysis by continuous discussion to ensure that there is alignment across the entire dataset as well as the coded extracts.
5. Definition and names of themes	Iterative discussions and analysis to further refine each theme in terms of the names and narratives that they convey about the research.
6. Production of the report	Using appropriate, rich extract examples and checking that the analysis relates to the research question and the literature to ensure that the qualitative research is well presented in any reports. The study findings will be tested in the quantitative health economics phase of the study.

Procedural precision was addressed by maintaining an electronic logbook and memos of scheduled interviews and decisions taken in software analysis; data management on multiple password-protected platforms that received ethical approval and being able to demonstrate procedural logic to support study findings.

Finally, the following techniques were used to enhance the quality of the research [45, 46]: data collection over an extended period of time (prolonged engagement) contributed towards the trustworthiness of the findings (credibility); the transferability of the findings in relation to literature from other settings were debated and discussed; the dependability of the findings were enhanced using saturation and iterative data collection and analysis finally, peer debriefing that included discussions on potential biases, including our own, as well as previously described techniques contributed towards the confirmability of the findings.

Additional information regarding the ethical, cultural, and scientific considerations specific to inclusivity in global research is included in the S1 Checklist.

## Results

In total, 49 participants were interviewed (28 of whom were female). Table 2 describes the profile of study participants. Interviews were discontinued once saturation was reached per study site (Table 2).

**Table 2:** Profile of study participants.

Study site	Number of academic participants	Number of medical participants (junior*; senior**)
A	6	6 (0; 6)
B	5	6 (1; 5)
C	5	5 (3; 2)
D	5	11 (6; 5)
**Total**	21	28 (10; 18)

* Junior doctors are those with less than two years of experience since graduation

** Senior doctors are those with more than two years of experience since graduation

The analysis generated 257 codes that were grouped into 24 categories, which subsequently formed the three themes and corresponding sub-themes. Two overarching themes were developed: *We know* and *We hope*.

### Theme 1: We know

Table 3 outlines the first theme with the related sub-themes.

**Table 3:** We know.

Theme 1 The certainty of issues in the medical curriculum: “We know”
**Sub-theme**
• That our institutional and academic processes are not satisfactory
• That there are negative experiences with hierarchy
• That there are consequences for the absence of formal management training
• That we need to teach management intentionally and explicitly
• That the formal inclusion of health economics is not critical
• That research training is important

### [We know] That our institutional and academic processes are not satisfactory

Both academic and medical participants described the constraints (real and perceived) of medical schools in fully incorporating management and research, and to a lesser degree, health economics in a curriculum that is dominated by clinical disciplines. The teaching and assessment of management and health economics was generally reported to be non-existent or ad hoc and unstructured. The inclusion of particularly management was often juxtaposed against the curricular time constraints with: *“…very*, *very important for it to be taught in the medical curriculum*. *But…you know*, *the medical curriculum is so packed we definitely can’t put everything*.*”* [Academic, SS B]. Educationalists were wary of the opportunity cost incurred to include teaching of management that would reduce the time for clinical training.

Medical schools were viewed as not being able to fully prepare students for the multiple realities due to amongst other factors, perceived staff and time constraints but there was recognition that it was vital to teach students about the management of self *“…but for me the one thing that we need as doctors is self-awareness*. *To be aware of what we can and cannot do*. *Because the perception out there is that…in my language we say*, *(makgona tsotle—you can do anything and everything) …and we can’t*.*”* [Academic, SS B].

Without the explicit inclusion of particularly management in the curriculum and being sheltered from decision-making in authentic situations, medical participants also spoke about the dramatic shift required with decision-making that happened once they graduated *“…because when we are undergraduates*, *we really don’t have much responsibilities…because everything will always be upon our seniors…a senior is expected to have those qualities and to even put them in practice*. *But they forget that we don’t even learn that from undergrad* [studies].*”* [Senior doctor, SS A]

### [We know] That there are negative experiences with hierarchy

Medical participants spoke about the power differential experienced in lecture halls during their undergraduate studies and to a large extent attributed this to the lack of teaching of management–especially around agency and self-regulation. Participants suggested that the development of agency would countermand this reality to some extent: “*…we’ll never get rid of a power differential in a hierarchical system where you have consultants*, *registrars and intern students*. *There is an inherent power in that system*. *But agency is something that I do think that we should be developing in students…their own sense of agency*.*”* [Academic, SS C]

Medical participants also testified that their negative undergraduate experiences with hierarchy continued after graduation and appeared to intensify with *“When you’re an intern you’re like the bottom of the food chain*. *And you’re there to just do the work (laughs)…But even as a medical officer*, *ja*, *voicing your concerns is something you have to do repeatedly before someone actually acknowledges or sits down with you and says*, *okay*, *let’s make a plan*.*”* [Junior doctor, SS C].

Finally, participants reported that society reinforces the notion of hierarchy—that the doctor is the individual to lead and manage healthcare team situations: *“…it’s not like medicine is a pyramid and we’re sitting at the top*, *but people do look to us as leaders*. *Patients kind of give us that role as well*.*”* [Senior doctor, SS D]

### [We know] That there are consequences for the absence of formal management training

The shortcomings of the undergraduate curriculum in preparing doctors for situations requiring management skills were discussed. In particular, participants from both groups who had medical training reflected on incidents and career choices where having any prior formal management training could have changed the outcome or experience for the better.

*“I think if…I knew more about management…I would have been able to plan things better*. *Things like preventive interventions at the hospital needed to be organised*. *I didn’t really have the tools…I did that [work] for ten years*. *I had no clue what I was doing in terms of management*, *you know*. *So*, *there I also felt a great absence of those skills*.*”* [Academic, SS C]

Medical participants also recalled that there was an emphasis on clinical training in the curriculum to prepare future doctors for the management of life-threatening medical incidents that they will encounter, but that there was no similar preparation for the management complexities that they came across, despite the possible impact on service delivery. Medical participants also expressed regret at not taking advantage of formal ad hoc teaching opportunities that were presented but acknowledged that as students, their focus was on clinical learning.

*“Medical students would not…be thinking about management and leadership*…*even right now*, *I would have probably not be willing to pay for it…regardless of how much it is*. *And if you made it free*, *you’d probably have to make it compulsory…I think we were just more like children thinking about how to attend to the patient rather than how to be leaders*. *It was mentioned…certain aspects were mentioned about leadership and such*, *but I think our brains were more wired to learn and think about certain things and this was not part of them*.*”* [Senior doctor, SS A]

All participants shared in detail–and some very emotionally—at least one experience where they felt unprepared and overwhelmed when management of self and others were needed.

*“I think it really happened a lot when I was an intern*, *because when you’re an intern and you’re posted at a district hospital*, *where there’s severe shortage of staff*, *you are given a ward*, *or even two*, *to manage…I felt I had difficulty*, *coming in as a new doctor*, *expecting there to be like three levels of doctors*, *medical officer*, *senior medical officer*, *resident*, *and specialist*, *and then I’m taken to a district hospital where there’s just me*, *the intern…It took a lot out of me going*, *waking up and realising you are the manager here*, *there’s no one else*.*”* [Senior doctor, SS A]

### [We know] That we need to teach management intentionally and explicitly

The (incorrect) perception by students that a doctor only needs clinical competencies was a challenge that would have to be addressed. Participants agreed that only focussing on clinical content does not meet the societal expectations of a medical doctor or the health needs of the population and suggested ways to capitalize on current opportunities in the curriculum and create new ones such as a management rotation for interns, industry partnerships to enhance exposure to the private sector platforms of managed care and health facilities, multi-rater feedback for senior students with regard to learning more about their leadership styles with peers and supervising clinicians and experiencing a typical day of a manager, consultant or chief executive officer in the health industry.

Medical participants acknowledged the challenge of teaching managerial skills, especially in the often didactic style that they experienced: *“…unfortunately when it comes to PowerPoint teaching*, *they don’t actually teach you the real life scenarios of what’s happening* …[but] *it’s better to be taught*, *it’s better to have half of something than nothing at all*, *that’s what I think*…*it’s better to be taught that than just to be pushed into the work environment and then you have to learn as you go*.*”* [Senior doctor, SS A]

The need to have more authentic experiences that would better reflect what awaits them was stated with *“I think some aspects like teamwork*, *sure*, *we did like little group activities and special study modules*, *sort of thing*, *where you have to work with a team and to write an assignment…it’s different when you have to write something like if someone doesn’t carry their weight with writing a research protocol*, *it’s not quite the same as if like you’re working as an intern and someone shows up late every day*.*”* [Junior doctor, SS D] The common strategy of students who fill the gap to compensate for a poorly-performing team mate was clearly not an option in the workplace.

Participants from both groups were cognisant that the timing of the management content in the curriculum needed to be ingrained early on with a focus on self-management that would signal to students the importance of it, albeit that they would apply it later in their career: *“So I think if it had been started earlier*, *as we finish we finish with that mind-set of knowing*, *I’m not only a clinician but I’m also a manager…I’m going to be working with staff that are junior than me*.*”* [Senior doctor, SS A]

### [We know] That the formal inclusion of health economics is not critical

The teaching of health economics was considered by participants with medical training to be a *“nice to have”* [Senior doctor, SS B], and unlike management and research, was not regarded to be essential in an undergraduate medical curriculum. Despite the use of a descriptor card and clarification about the scope of health economics, the focus of discussions in interviews was often around the role of budgets and financing in health systems, resource management and personal financial literacy that form elements of health economics but cannot claim to constitute the core of it *“…so understanding the cost associated with every test*, *exam*, *that we do*, *with every piece of paper that we are writing on*, *to understand that there is a cost to it*, *would be beneficial in terms of managing resources*.*”* [Senior doctor, SS A]

### [We know] That research training is important

All the participants acknowledged that research training is important to further develop evidence-based practise in students. All the study sites had formal research teaching. Although variation in the teaching was described, it was reported to be acceptable and of a high quality by some participants.

*“It was well structured*, *we were taught how to carry out research*, *we even had a project where we had to carry out a research and that was assessed as well…we could also see its importance even when we moved into the clinical phases of our internship…We even managed to get other skills from there*, *like doing clinical audits which is basically a subset of research …”* [Senior doctor, SS A]

The research skills to generate research questions and retrieve and appraise literature to inform a decision for evidence-based practise was considered important because *“…if they do not have that knowledge they will practise medicine based on their experience*. *Experience is good but experience is not enough*. *Experience will need to be backed up by evidence and that evidence comes from the literature*.*”* [Academic, SS B]

The common-held perception that a dichotomy exists between being medical doctor or a researcher was addressed by an academic participant who reflected on the role that research plays in any discipline.

*“…in the end it’s not about research…it’s about building good clinicians*. *But I also think being good clinicians is not necessarily mutually exclusive from being at least a decent researcher… I think research is how you really bridge that gap between practice and contemporary methodologies*, *contemporary strategies*, *or contemporary practices*, *so to speak…but at least a decent enough researcher that they can review the literature and use it; they can do audits in their context and being to really explore and understand the challenges that they might be facing*.*”* [Academic, SS A]

Medical participants also acknowledged that, for a number of reasons, they did not pay too much attention to the formal opportunities regarding research.

*“And I think if you ask me this question before I graduated*, *I would probably have said like*, *please let’s do less research*, *like scrap it off the curriculum entirely because I…hated research as a student…*, *But now I’m like*, *I wish they forced us to do more research because I’m like*, *it’s so relevant now and it’s something that everyone has to be able to do…And I’m only realising now how few of us actually know how to do research*.*”* [Junior doctor, SS D]

Incentives for additional marks or credits and opportunities to become more involved in undergraduate research societies where offered were seldom pursued. Almost all of the medical participants expressed regret in hindsight.

*“…You know*, *if I look back now I wish I would have done that but I wasn’t one of the top students*. *I had to dedicate my time to my studies and that so I didn’t actually have that mindset at the time to do it*. *I don’t think I actually understood the benefits of it*. *It would have been nice to have that research behind my name now applying for job and for registrar programmes*.*”* [Junior doctor, SS C]

### Theme 2: We hope

Table 4 outlines the second theme “We hope” with the related sub-themes.

**Table 4:** We hope.

Theme 2 Acknowledgement about the sub-optimal training of management and research but hope that medical doctors will still be able to fulfil expectations: “We hope”
**Sub-theme**
• That our medical graduates would be skilled in using research
• That our medical graduates will exhibit the managerial competencies when required
• That our medical graduates will have voices for others

### [We hope] That our medical graduates would be skilled in using research

Participants acknowledged the deficiencies in the research training and desire for the curriculum to be optimised, culminating in graduates being more effective medical practitioners. Academic and medical participants wished to optimise the research training, especially in relation to the duration of allocated time; the timing in the curriculum and the learning outcomes. The allocated time was considered as inadequate and reinforced the (unconveyed) importance of research in medicine.

*“…I don’t think eight weeks is enough for us to really grasp the skills as well as the knowledge necessary behind it*. *Because more often than not we’re just there as students*, *undergrads*, *you’re just cutting corners*, *just make sure that you meet the deadline*. *So then in between messages get lost*. *You are just doing things because now we just have to*, *you just have to*, *you don’t really understand why you need to…But you haven’t really given me enough time to grasp the concepts*.*”* [Senior doctor, SS A]

There were also suggestions to review current learning outcomes to link research to medicine *“I think it’s very important for the average medical student*, *must at least know how to appraise an article …some of the articles out there are not of good quality*, *and to base your decisions*, *your clinical decisions on an article that has poor evidence is very dangerous in terms of affecting patient well-being*.*”* [Junior doctor, SS D]

Creating a culture of research at institutions where undergraduates could see and appreciate its role in their chosen careers, was emphasised with “*I think the best thing a university could do*, *or a curriculum can offer*, *with something like this*, *is a culture*. *There needs to be a culture with like*, *you know*, *this is where actual medicine comes from*, *it’s from research*, *it’s from looking at all the latest things*.*”* [Senior doctor, SS D]

Medical participants expressed the need that medical schools be explicit about the importance of research skills, especially for career paths.

*“…the incentive for doing research*. *Which has become more prominent now that I’m like needing to apply for an MO* [medical officer] *post and preparing to apply for a registrar post…it counts for so much in terms of how competitive it is to get posts*, *and in terms of your personal development and like your CV at the end of the day*.*”* [Senior doctor, SS D]

There were also educationalists who supported introducing qualitative research in training with *“And I think also to look at qualitative data*, *because I think a lot of times medical specialists are so focused on the quantitative research that they forget that qualitative research can give them so many answers to what their patients are experiencing*.*”* [Academic, SS C].

Participants from both groups were supportive of intercalated research degree programmes in any field as they recognized that, although a minority, that there are medical students who are exceptional and would be able to do dual studies as *“…there are just bright students out there that can manage to do both and that can propel them forward if they did another degree*, *whether it be in economics or in epidemiology or whatever it is*. *And for those who see themselves in doing more than just the narrow practice of medicine*.*”* [Academic, SS C]. However, the costs of developing this exceptional programme should be weighed against the possible benefits of strengthening the research training for all medical students.

The assertion that medical doctors need to know how to use research as opposed to doing research was expressed by several participants. Different research objectives were also crystalized by academic participants:

*“But I think it’s important that a doctor learns a few things*. *One*, *they understand the ethics of research*. *Two*, *they understand the responsibility of the researcher to the participants of research*. *And three*, *that eventually a doctor may not necessarily turn out to be a researcher*. *But they can evaluate evidence*, *and that is a skill*…*And so*, *being able to evaluate evidence*, *and…and then deciding which one to use and which one to know*, *and not necessarily apply in their context*.*”* [Academic, SS A]

Finally, although publication at undergraduate level was supported, it was unequivocal that it should be optional as: *“…I don’t necessarily think that expecting them to publish should be the core rule; it should be encouraged if they can*.*”* [Academic, SS A]

### [We hope] That our medical graduates will exhibit managerial competencies when required

Academic participants reported a reliance on the formal teaching of clinical content to, in some way, instil values, habits and best practices of management: *“We sort of hope that by taking part in these practical exercises they will pick up something about management and leadership*.*”* [Academic, SS A] and *“You kind of hope that a student during the course of a block somewhere hits on one of those people and gains their leadership skills from that*.*”* [Academic, SS D].

### [We hope] That our medical graduates will have voices for others

Despite not being explicit about management competencies and its importance, academic participants expressed a fervent hope that medical doctors will develop the prerequisite knowledge and skills to be able to champion the causes of those less fortunate than them.

*“So now being an advocate of health is nowhere in the curriculum but as a medical doctor it’s one thing that is important*, *because you need to advocate for the people who are in the community where you practise*, *who are going to benefit from your services and your skills…you may not be able to give them some things*, *but you can probably use your status to…get some of those problems solved*.*”* [Academic, SS C]

## Discussion

This study explored the opinions and experiences of medical doctors and academic educationalists on the inclusion of management, health economics and research in the medical curriculum. Although studies have demonstrated that more effective practitioners (the so-called twenty-first century physician leader) have attributes related to management and research [15, 47], there is still repeated emphasis on the inadequate, unstructured and/or ad hoc teaching of management and research in the curriculum by academics and medical students [17, 23, 48].

**Fig 1:**
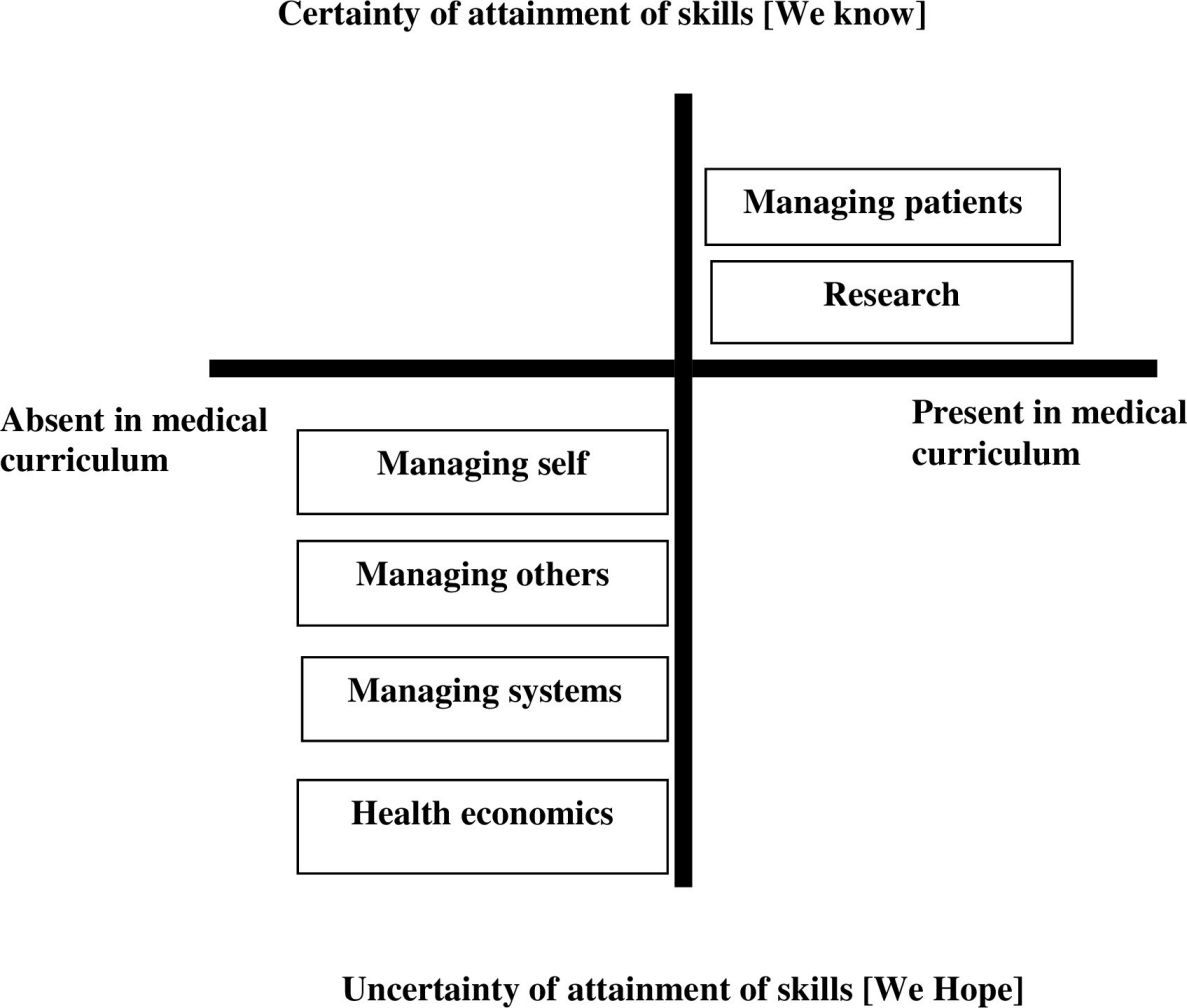
Typology of study findings.

A typology that emerged from the findings of both themes is illustrated in Fig 1. The degree of certainty that both participant groups had about the presence of identified topics in the medical curriculum was juxtaposed with the degree of confidence about the attainment of the skills related to these topics. For example, the management of self, others and systems was to a large extent absent from the curriculum but it was still hoped that medical graduates would somehow attain the competencies when placed in situations that required them.

The majority of participants with medical training described pre-pandemic impacts on their wellbeing and clinical practice when they transitioned from being students to medical doctors. These impacts include the inability to manage or prioritise self-care, at times resulting in burn-out; the inability to make or implement management decisions in the workplace with confidence and the career limitations encountered by not knowing about the importance of research. All participants shared that having any formal training on managing themselves, working with others and more opportunities to develop skills to understand and navigate the health system would have been of benefit.

Regarding research, the limited time allocated to training was noted as a discouraging factor that contributed towards the research inexperience of most medical participants. This was consistent in other studies [23, 25]. Institutions that have used authentic alternatives to instil evidence-based medicine and research skills use both extra-curricular and curricular platforms [30]. The strengthening of current research training by incorporating research skills in clinical rotations towards evidence-based practice was a common recommendation. Involving students in journal clubs and academic research project discussions would also cultivate a research culture and convey the importance of these skills in their careers [49, 50].

In comparison to the management and research study findings as well as health economic literature in the medical curriculum, this study found that support for the formal inclusion of health economics in the curriculum was insignificant [21, 24, 41, 51]. Participants instead supported the inclusion of health economics as an optional elective offering for those who were interested. in postgraduate studies. One possible reason for the different finding to what has previously been reported in the literature are that the study setting as the participants worked to a large extent in environments where they were less likely to be engaged in decision making on drugs and treatments with patients in comparison to a setting such as the National Health Service in the United Kingdom [39]. Another possibility is the view that the discipline of health economics would require extensive teaching with limited practical application opportunities and would be at the expense of other subjects.

There was regret expressed by participants that they did not take advantage of known formal or ad hoc opportunities to engage with these topics. This is balanced by the notion that with staff and curriculum time constraints, there is a bounded rationality that we can only present students with a limited number of opportunities to learn for multiple realities that may or may not occur [52]. However, similar to the health systems science curriculum framework that is presented by Borkan et al. (2021) with core and cross-cutting domains, there is a recurring need for the medical curriculum to go beyond the clinical content and prepare future doctors with appropriate skills to better serve populations under their care [13].

Finally, the findings of this qualitative research study allude to the formal inclusion of particularly management and research content in the curriculum but also their prioritisation in with “*…but being very careful to balance the equation and not do it at the expense of the clinical training*, *which is also vast in its length and depth*.*”* [Academic, SS B] This raises the question of what clinical teaching time individuals are willing to give up for research and management training. Future research will apply the discrete choice experiment (DCE) methodology to explore this. Whilst DCEs have been used extensively to value attributes (or characteristics) of health and health care, to date they have not been used to inform optimal curriculum design [53, 54]. In this instance, a DCE will be used to inform optimal curriculum design by presenting respondents such as medical doctors with a series of medical curricula choice tasks (a set of options characterized by various attributes). For each choice they will be asked to select the curriculum option that they prefer. Based on the qualitative study findings, the attributes will include clinical training time, management and research content and the timing in the curriculum that the content may be presented to students. Due to the significant change that the pandemic had on the teaching methods in institutions, an attribute about different teaching methods in relation to the management and research attributes may also be incorporated. Ultimately, the DCE will allow us to estimate how much clinical training time respondents are willing to give up for the teaching of management and/or research content.

The choice of study design and the qualitative nature of the research has their inherent limitations. Conducting this study at the start of the COVID-19 pandemic meant that the most of the interviews were conducted virtually, amidst competing priorities for the time and attention of participants. This may have influenced the depth to which the need for management and research skills was discussed.

## Conclusion

Despite acknowledgement that management and research are topics that need to be taught, educationalists appeared to rely on chance to teach and assess management in particular. These qualitative study findings will be used to develop a DCE to inform optimal curricula design.

## Supporting information

S1 Checklist (DOCX)
